# Role of Implantable Drug Delivery Devices with Dual Platform Capabilities in the Prevention and Treatment of Bacterial Osteomyelitis

**DOI:** 10.3390/bioengineering9020065

**Published:** 2022-02-06

**Authors:** Caroline Billings, David E. Anderson

**Affiliations:** College of Veterinary Medicine, University of Tennessee, Knoxville, TN 37996, USA; dander48@utk.edu

**Keywords:** bacterial infection, osteomyelitis, drug delivery device, biofilm, nanotechnology, tissue regeneration, antibiotic, antimicrobial drug

## Abstract

As medicine advances and physicians are able to provide patients with innovative solutions, including placement of temporary or permanent medical devices that drastically improve quality of life of the patient, there is the persistent, recurring problem of chronic bacterial infection, including osteomyelitis. Osteomyelitis can manifest as a result of traumatic or contaminated wounds or implant-associated infections. This bacterial infection can persist as a result of inadequate treatment regimens or the presence of biofilm on implanted medical devices. One strategy to mitigate these concerns is the use of implantable medical devices that simultaneously act as local drug delivery devices (DDDs). This classification of device has the potential to prevent or aid in clearing chronic bacterial infection by delivering effective doses of antibiotics to the area of interest and can be engineered to simultaneously aid in tissue regeneration. This review will provide a background on bacterial infection and current therapies as well as current and prospective implantable DDDs, with a particular emphasis on local DDDs to combat bacterial osteomyelitis.

## 1. Introduction

### 1.1. Implantable Medical Devices—Benefits and Challenges

There are continual advances made in the fields of medicine and science. These advances include the creation of a wide array of implantable medical devices from indwelling vascular or urinary catheters to total hip replacements and cardiac pacemakers. Each of these devices serves a unique purpose, and despite significant differences in form and function, implantable medical devices are uniformly considered to improve the quality of life of the patients in which they are utilized [1]. Approximately 8–10% of Americans, or 5–6% of people in industrialized countries, are estimated to have received an implantable medical device [1,2]. In the United States alone, there are more than five million medical devices or implants used annually [3]. Implantable medical devices serve a wide variety of indications and allow physicians to improve patients’ lives by stabilizing complicated fractures using metal rods, pins, screws, and plates [4,5]; providing children suffering from sensory deafness with practically normal speech and language development by utilizing cochlear implants [1,6]; and providing high quality of life to patients suffering from cardiac disease by implantation of artificial valves [7], pacemakers [8], and cardiac defibrillators [1,2], among many other procedures. Despite incredible advances in the impressive arena of implantable medical devices, there remain persistent challenges. These challenges include insufficient biocompatibility of devices, which can be associated with foreign body responses [9,10,11], biofilm formation on devices [3], and chronic bacterial infection associated with the site of implantation or device [11,12,13,14]. Each of these challenges may lead to implant failure at any point during the in situ lifetime of the device [15].

### 1.2. Bacterial Infection—Risks and Current Therapies

Bacterial infection is a common, yet catastrophic, complication [16] that can occur following the implantation of a medical device, regardless of body site [11,13,17]. Bacterial infection falls under the umbrella of healthcare-associated infections (HAIs) [18]. The Centers for Disease Control (CDC) report that surgical site infections (SSIs) comprise 20% of HAIs, with an estimated 110,800 SSIs reported in 2015 [19] and an estimated one million implant-associated infections occurring each year [20]. Average rates of infection for initially inserted implants range from 2 to 40% depending on implant type, with orthopedic implants in the range of 2–5% [21]. Bacterial infection can be caused by traumatic or contaminated wounds, such as open fractures [12,16,22], as well as hematogenous or perioperative bacterial seeding [23]. The presence of an implant increases the risk of infection [24]. The risk of infection is multifactorial and thought to be due in part to lowered local host defenses [12,13,25] as a result of tissue trauma, presence of foreign material [13], and alterations in fluid dynamics [26]. These described alterations, in combination with the trauma of disease and device placement, can create a local environment that is susceptible to infection with a lower infective dose of bacteria [12,13,25,27]. Additionally, bacteria may be able to adhere to indwelling devices and form three-dimensional communities of bacterial cells and exopolysaccharide matrix (biofilms). These biofilms can cause persistent, recurrent bacterial infection and are typically resistant to traditional antimicrobial therapy [3,13,17,20,21,28,29,30,31]. Separately from implant-associated complications, host factors such as systemic health and lifestyle play a role in the manifestation of bacterial infection. Examples include patients with comorbidities such as diabetes mellitus, obesity, immunosuppression, and patients with certain lifestyle choices such as smoking [12,32,33,34,35,36].

Current treatment for implant-associated bacterial infection is comprised of prolonged systemic antimicrobial therapy (often lasting weeks to months), revision surgeries, and implant removal. Revision surgeries include local debridement of compromised tissues and, typically, removal of the associated implant followed by re-implantation of a new device after resolution of the infection [21,25,37]. Re-implantation of a new device prior to complete clearance of infection can reinitiate the process and add to a prolonged and difficult recovery. These cases often require multiple revision procedures and are accompanied by risk of failure at each step [25,38,39], as well as a higher risk of infection than initial implantation [40]. These treatments can increase the costs of healthcare and may require hospitalization of the patient. Another area of concern is prolonged systemic antimicrobial therapy, which can be accompanied by adverse systemic side effects or toxicity, as well as increased risk of antimicrobial resistance [11,20,41,42]. These cases are challenging for clinicians to successfully diagnose and manage [21,25,39,43,44]. They also place a heavy burden upon the healthcare system, as management often extends the duration of hospital stay [45] and is expensive, with cost estimated to range from $10,000 to $25,000 per case, depending on the type of implant, degree of infection, and treatment protocol [21,34,45,46,47]. Costs may increase by $20,000 per admission into the hospital [19], may exceed $90,000 when a prosthetic joint is involved [47], and may exceed $150,000 if following orthopedic trauma [48]. Important to note is the physical and psychological impact of implant failure [21,43] and treatment protocols on the patient. Undoubtedly, the treatment process has a significant impact on the patient’s quality of life [34,44,49], and this should be kept in consideration when undergoing diagnosis, surgical planning, and management of implant-associated bacterial infection. The focus of this review is to describe the current approach to bacterial osteomyelitis and the use of drug delivery devices in the management of this disease.

### 1.3. Bacterial Osteomyelitis

Osteomyelitis is an inflammatory disease caused by infecting microorganisms that leads to bone destruction and, ultimately, progressive bone loss [50,51,52]. Typically, osteomyelitis is caused by bacteria, most commonly by Gram-positive *Staphylococci* species including *Staphylococcus aureus* and *Staphylococcus epidermidis* [50,53,54], although fungal osteomyelitis occurs as well [53,55]. There are three main categories of osteomyelitis, listed here in order of decreasing frequency: (1) secondary to contiguous focus of infection, often resultant from trauma, surgery, or implanted prosthetic material; (2) secondary to vascular insufficiency, often a result of diabetic foot ulcers; and (3) hematogenous [44,53]. There is considerable variation in the etiology and presentation of osteomyelitis [45,50,52,53]. In this review, the focus will be on bacterial osteomyelitis that occurs following trauma, surgical procedures, or implanted materials, which is reported to account for 47 to 50% of osteomyelitis cases in adults [50,52]. These cases are most commonly caused by *S. aureus* [44,45,55,56,57,58,59], including methicillin-resistant *S. aureus* (MRSA) [55].

Osteomyelitis may present acutely with fever, pain, abscess formation, signs of local inflammation, and a draining tract. These symptoms of osteomyelitis may present shortly after reduction of a traumatic, open fracture [50,51,53], or other surgical procedure. While this presentation is certainly compelling for active bacterial osteomyelitis, confirmatory evidence may not be present on diagnostic imaging such as radiographs for 2–3 weeks following onset of infection [52,55]. In contrast, patients suffering from chronic osteomyelitis resulting from presence of avascular, necrotic bone, biofilm, or prosthetic material may present years after the initial insult [34,60,61]. These cases are more likely to present with a subtle constellation of symptoms and may be recognized solely by focal tenderness during physical exam [44,53,62], with suspicions increasingly raised on diagnostic testing [63,64]. The distinction between acute or chronic osteomyelitis is often challenging and may require histopathological examination of bone biopsies to delineate disease chronicity at the cellular level [36,62,63,65]. Histology may also demonstrate the presence of both chronic and acute changes in a single specimen [65], highlighting the progressive nature of osteomyelitis. These variations contribute to the challenge of obtaining a swift and specific diagnosis of osteomyelitis. Diagnosis typically involves physical examination, radiographic imaging, hematology and serum biochemistry, culture of blood, and wound tissues, and often includes advanced imaging such as computed tomography (CT) or magnetic resonance imaging (MRI) [51]. The gold standard for diagnosis involves bone biopsy and culture [36,44,52,53], as well as histopathological examination of the bone [34,36].

Once diagnosed, bacterial osteomyelitis should be treated intensively, as osteomyelitis increases patient mortality by an estimated 8% [45]. Treatment for this type of osteomyelitis is similar to that described above for general bacterial infection, as it is constituted by surgical debridement of affected tissues, removal of affected implants or material, preservation of vascular supply, and systemic antimicrobial therapy guided by culture and sensitivity [25,32,34,44,51,52,53,65,66,67]. The success of osteomyelitis therapy lies within the quality of surgical debridement [50,68], which is challenging because of the need to debride all affected tissues while also preserving as much form and function for the patient as possible [25,32,44]. Inadequate debridement is one of the most common reasons for reoccurrence of chronic osteomyelitis [44]. In addition to thorough debridement, antimicrobial therapy is required and is accompanied by challenges such as inadequate penetration, antimicrobial resistance, presence of biofilms on devices or necrotic bone, and side effects of protracted antimicrobial therapy [44,45,56,66,69]. When an implant is associated with osteomyelitis, the decision to leave or remove the implant [17,65,69] is crucial and can impact treatment and patient quality of life. Additionally, replacing an implant following debridement and antimicrobial therapy poses a challenge, as locally compromised host defenses will render that site more highly susceptible to bacterial infection [13,25,70].

Major challenges in osteomyelitis therapy include antimicrobial therapy and inadequate penetration into bone, risk of chronic or recurrent bacterial infection, and extensive tissue destruction [34,60,65,66]. These challenges can be attributed in part to the abilities of *S. aureus* as a pathogenic organism [55,61]. *S. aureus* is not only a common commensal species [71,72], but also a versatile competitor and dangerous pathogen, with virulence factors that lend themselves towards causation of a diverse range of diseases [73,74,75]. The ability of *S. aureus* to evade the immune system adds to the difficulty of effectively treating osteomyelitis. Immune system evasion is accomplished with four main mechanisms, including the following: (1) Abscess formation [65,76]. This is a process controlled by the host and pathogen. This process ultimately offers protection to *S. aureus* by sequestering a focus of infection away from easy immune system access [65,77]. (2) Biofilm formation [36,76,78,79,80]. Biofilms offer protection by providing a physical barrier between immune cells and bacterial cells, while providing immense phenotypic diversity, which lends itself towards antimicrobial resistance. Biofilms also allow for horizontal gene transfer and acquisition of virulence mechanisms [65,76,81]. (3) Osteocyte-lacuno canalicular network (OLCN) invasion [65,76,82]. The ability of *S. aureus* to gain access to the canalicular system of bone is thought to provide nutrients to bacterial cells while simultaneously protecting these cells from immune attack. OLCN invasion is proposed as an important mechanism in the persistence and recurrence of osteomyelitis [82]. (4) Intracellular persistence of *S. aureus* [36,49,65,80,83]. *S. aureus* has proven to be capable of internalization in a variety of cell types including osteoblasts [83,84,85,86,87] and, although the mechanisms are not fully understood, any period of intracellular persistence is thought to protect *S. aureus* from the immune system as well as antibiotic therapies [76,83].

With these challenges in mind, as well as the desire to reduce financial burden, length of hospital stay, and patient morbidity and mortality [45,65,88], the need for effective, practical strategies to improve the therapy of osteomyelitis is clear and leads investigators to study local drug delivery devices (DDDs), especially those that can serve as tissue regeneration platforms [44,56]. Local administration of antimicrobials can be accomplished via biodegradable or non-biodegradable DDDs. Degradable devices are desirable to eliminate the need for future removal of the device. Local administration is intended to mitigate the side effects of systemic antimicrobials while providing greater local concentrations of antimicrobials [56,66,89,90,91,92] and improved penetration to target tissues [41] to more effectively clear bacterial infection [93]. Recently, drug delivery systems have been designed to possess dual platform capabilities to aid in bone regeneration [56]. This class of multifunctional devices holds immense promise in the treatment and clearance of bacterial osteomyelitis and offers to improve the lives of patients suffering from this life-altering disease.

## 2. Desirable Characteristics of Local Drug Delivery Devices

There are many local drug delivery systems that have been investigated [9], and through these investigations, certain characteristics have emerged as most beneficial to success of the system. Ideal characteristics include the following:

1. Biocompatibility [2,56,94,95,96];
2. Predictable, inert degradation [22,56,89,97];
3. Sustained, clinically significant drug release [45,57,68];
4. Appropriate mechanical strength to support surrounding tissue [68];
5. Appropriate architecture to facilitate tissue ingrowth, when applicable [98,99,100].

In combination, these characteristics provide the conceptual ideal local drug delivery system. This system can load and elute antimicrobials at a clinically significant concentration, and for a suitable length of time, be safely implanted into the area of interest without causing a foreign body response [10]. This system will also degrade over a predictable length of time without generating harmful byproducts. The additional benefit of appropriate mechanical support is not provided by most of the currently investigated DDDs, but when available, can reduce additional surgical manipulations with hardware placement and lessen the risk of infection and device failure. Although these characteristics are universal to local DDDs designed to clear bacterial infections in multiple tissues, the examples in this review are specific to bone and the treatment of bacterial osteomyelitis.

The above description of sustained, clinically significant drug release is easily stated, yet difficult to fully understand and achieve [101]. In fact, drug elution kinetics in local DDDs have considerable variation [92] based on the size, shape, and composition of the delivery device [57,92]; the drug that is loaded [102,103]; the manner in which the drug is loaded [104]; and the local environment into which the device is placed [57,102,105]. To this end, in vitro testing of each device and drug combination must be completed prior to consideration for in vivo use. While an imperfect predictor of in vivo performance, in vitro testing is useful and may bring significant hurdles or advantages to light. Drug elution kinetics in traditional drug delivery systems rely heavily upon local flow rates, which often result in initial burst-release of drugs [57,93,103,104,106] from the material surface, followed by sustained, gradually reducing drug release [93,103] as the porosity of the device is exploited [101]. This drug release profile can be undesirable in vivo if drug release cannot be sustained over the minimum inhibitory concentration (MIC) and the minimum biofilm eradication concentration (MBEC) of the bacteria of interest [107], and if release cannot be sustained for a period of 3–4 weeks [103,104]. An attraction to stimuli-responsive materials is the controllability of drug elution kinetics [104], which may provide drug release over MIC for longer periods of time compared with traditional systems.

## 3. Local Drug Delivery Devices for Bacterial Osteomyelitis

### 3.1. Bone Cement

**Figure 1:**
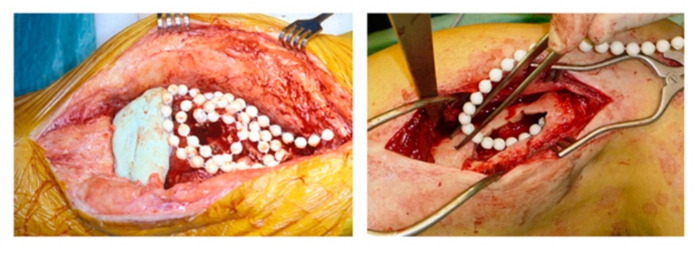
Antibiotic-loaded PMMA beads utilized in orthopedic procedures. In the left-hand image, PMMA beads are used in the treatment of total knee arthroplasty; in the right-hand image, they are used in the treatment of chronic osteomyelitis. Reprinted from van Vugt et al. [112] following the Creative Commons Attribution (CC BY) license (http://creativecommons.org/licenses/by/4.0/) (accessed on 11 July 2021).

Historically, the most widely utilized local drug delivery device for combatting bacterial osteomyelitis has been antibiotic-loaded polymethylmethacrylate (PMMA), or bone cement [34,65,89,108,109,110,111]. In orthopedic procedures, antibiotic-loaded PMMA is often utilized to create beads or bead chains (demonstrated in Figure 1) to pack infected sites or as a cement applied prophylactically to prostheses [57,101,112]. This system is considered the current gold standard [66,113,114], has multiple commercially available formulations [107,115,116], and is often used to complement parenteral antimicrobial therapy [117]. This system has clear benefits including mechanical stability, suitability for use with numerous heat-stable antibiotics [111,112], release of metabolically active [114] antimicrobial compounds above the MIC of most common pathogens over a period of hours to days following implantation [112], and elimination of dead space from debridement [34] or wounds. PMMA has recognized shortcomings including the exothermic polymerization reaction during PMMA formation, which limits the use of heat-labile antibiotics [17,112,115] and creates concerns of tissue damage or necrosis [98,115]; wide variation in elution kinetics based on the type of bone cement; antimicrobial compound and mixing method chosen [101,114]; and sand incomplete release of antibiotics, which raises concerns for persistent low level antimicrobial release and subsequent antimicrobial resistance [14,70,107,109,118,119]. Finally, the lack of degradation of PMMA beads is a recognized shortcoming, as persistent foreign material may create wear particles and is an excellent substrate for biofilm formation [17,107,109,115,120], which can be a nidus of inflammation or initiate bacterial seeding to other sites [121]. Additionally, the body may mount a foreign body response to the indwelling material, and additional surgeries for removal of persistent beads are typically required [41,108,112,118,119].

**Figure 2:**
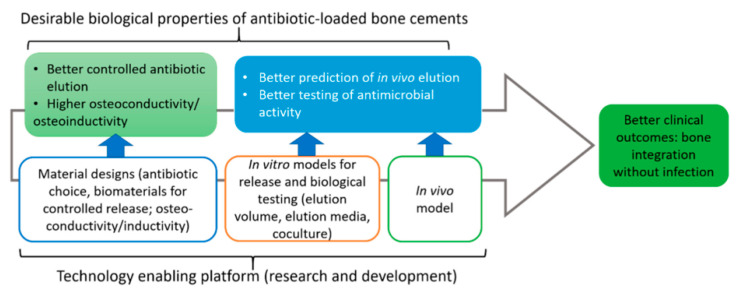
Flow chart describing the ideal properties of antibiotic-loaded bone cements. Reprinted from Wall et al. [115] following the Creative Commons Attribution (CC BY) license (http://creativecommons.org/licenses/by/4.0/) (accessed on 11 July 2021).

To improve PMMA as a local drug delivery system, there have been many investigations into the properties of bone cements, including antibiotic elution, bone ingrowth, and mechanical properties [115], as shown in the flow diagram in Figure 2. Porosity has come to light as an important factor [70] for both elution characteristics and bone ingrowth [98,122]. However, increased porosity can decrease the mechanical strength of the cement [31]. This highlights the need for balance in providing the desired porosity while maintaining sufficient mechanical strength. Frew et al. [123] investigated differences between in vitro elution characteristics of gentamicin and vancomycin from commercially prepared cement versus hand-mixed cement. They found that hand-mixing vancomycin powder into PMMA/gentamicin cement provided greater than a fivefold and twofold increase in cumulative elution of vancomycin and gentamicin, respectively. These results were accompanied by greater variation in elution as compared with commercially available prepared cement. Greater cumulative elution and wider variation in elution in this situation were attributed mainly to variation in porosity, as hand mixing is thought to create a more heterogenous mixture, which provides greater porosity and slightly poorer mechanical characteristics. These results align with the available literature, which cites mixing technique as an important determinant of porosity [70,124]. Nugent et al. [125] found that elution of tobramycin from PMMA increased with increased porosity, as caused by increased fraction of the poragen xylitol. They also discovered that the compressive strength of the cement decreased with increased porosity and prolonged elution time in vitro. Similar results are reported by other studies [110,126,127], and the loss of mechanical strength with additives is commented on by Arora et al. [128].

There have been investigations into the incorporation of bioresorbable, osteoconductive, or osteoinductive components such as calcium phosphate (CaP), tricalcium phosphate (TCP, α-TCP, β-TCP), and hydroxyapatite (HA) into bone cements with the goal of controlling antibiotic release and simultaneously encouraging bone ingrowth [98,115,129,130]. It has been determined that the optimal pore size for bone ingrowth is within the range of 150–400 µm [98,115], and CaP materials should be selected with that in mind [115]. Fini et al. [130] compared a PMMA and α-TCP composite with PMMA and found that the porous architecture of the composite increased osteoblast viability in vitro and had a significantly greater rate of new bone mineralization in rabbit bone in vivo. These results speak to the increased biocompatibility of the composite. Vazquez et al. [131] found that incorporating β-TCP particles into PMMA extended the curing time of the cement. This finding agrees with those of Beruto et al. [132] and Lin et al. [98], who found that the addition of chitosan/β-TCP microspheres to PMMA cement increased the curing time and decreased the curing temperature of the cement composites. These findings are helpful, as they offer the surgeon additional time to form the cement and provide a lower polymerization reaction temperature, which lessens potential tissue damage and may improve biocompatibility [98]. The option for a bioresorbable component to bone cement is intriguing and may eliminate the need to remove PMMA/CaP composites, as increased biocompatibility, osteoconduction, and osteoinduction may lead to device integration into bone [115].

Despite these investigations and advances with regard to antibiotic-loaded PMMA, challenges remain that raise concerns for the long-term suitability of this system for use in the clearance of bacterial osteomyelitis. In fact, leaders in the treatment of musculoskeletal infections met in 2019 for review and discussion of the available literature regarding antibiotic-loaded bone cement. They concluded that, although it is used frequently and supported anecdotally, there is a lack of strong evidence supporting the clinical benefit of this drug delivery system [107]. Persistent challenges include the precise tunability of elution kinetics, as this system is affected by many manufacturing variables and raises concerns regarding the predictable in vivo performance of PMMA [107]. Additionally, the risks of biofilm formation on indwelling PMMA, antimicrobial resistance from extended low level antimicrobial elution, and the need for surgical removal of this system are major [107]. These risks are unlikely to be completely overcome without severely compromising the mechanical properties or drug delivering capacity of this material. With these challenges in mind, there has been increased interest in biocompatible, biodegradable devices [109], which will be discussed further.

### 3.2. Bone Grafts

Autologous or allogenic bone grafts are sometimes utilized in the treatment of osteomyelitis, particularly when there is substantial bone loss, whether that is the result of trauma or extensive debridement of compromised bone [36,50,133,134]. While autologous bone grafts provide excellent osteoinductive, osteogenic, and osteoconductive properties, they require an additional harvest procedure, which is painful and can create donor site morbidity [66,90,135,136,137]. Recently, Kim et al. [135] proposed that the proximal tibia be utilized as a harvest site for cancellous bone, suggesting that this procedure is less painful than the traditional anterior iliac crest harvest site. Owing to challenges in harvest procedures and concerns of patient morbidity when large amounts of bone are required, allogenic bone grafting is sometimes elected instead of autologous grafts [66,137,138]. Allogenic bone grafts are typically used either frozen or freeze-dried, rather than fresh, to reduce the risks of immunogenicity or disease transmission [138,139]. While these methods are helpful to prevent rejection of the graft, the mechanical, osteogenic, and osteoinductive properties may be adversely affected by the processing [137,140,141]. In 2010, Ketonis et al. [142] reported that bone allografts are utilized for more than 800,000 musculoskeletal procedures in the United States each year. Unfortunately, over 11% of implanted bone grafts develop infection, which is thought to be due in part to biofilm formation on the implanted graft [142].

Regardless, autologous and allogenic morselized cancellous bone is used in various orthopedic applications and is proposed as a drug delivery device for the treatment of chronic osteomyelitis [56,136,142]. Cancellous bone can be impregnated with antibiotics before implantation either by mixing powdered antibiotics with the graft or direct soaking [57]. Lewis et al. [136] demonstrated rapid in vitro release of antibiotics (gentamicin) from bovine cancellous chips within the first two days after impregnation, followed by a consistent rate of release for the remainder of the 14-day study. Lewis et al. [90] also reported that demineralized bone matrix (DBM) could be loaded with, and deliver, gentamicin locally without diminishing the osteoinductive properties of DBM in an in vivo rat ectopic pouch model. Ketonis et al. [142] investigated the feasibility of bonding vancomycin to morselized allograft bone to mitigate bacterial colonization of the allograft. They found that vancomycin could be covalently modified and that *S. aureus* colonization was prevented in vitro. Covalent modification of antimicrobials presents an intriguing option to prevent biofilm formation on allografts.

Despite the intriguing experimental discoveries centered around bone allografts as multifunctional systems, it is unlikely that autologous or allogenic bone grafts will serve the need for a single system to achieve simultaneous treatment of bacterial osteomyelitis and bone regeneration. The described limitation is a reflection of the trade-off between desirable osteogenic capabilities and the risk of immunogenicity and infection that accompanies allogenic grafts [137,143], as well as the serious risks of patient morbidity that accompany autologous grafting [66]. Recognizing limitations with bone grafts as stand-alone local DDDs, the most promising option remains to incorporate these components into other systems. There have been extensive investigations into alternative materials to deliver antimicrobial compounds and provide the osteogenic properties necessary to regenerate bone tissue.

### 3.3. Synthetic Bone Graft Substitutes

Synthetic bone graft substitutes are of interest for local drug delivery in the management of osteomyelitis, especially because they hold potential for dual-platform functionalities. This classification of material includes ceramics such as calcium sulfate [108], calcium phosphate, and porous alumina [17,68,120,144], as well as bioactive glass [17,145].

#### 3.3.1. Calcium Sulfate

**Figure 3:**
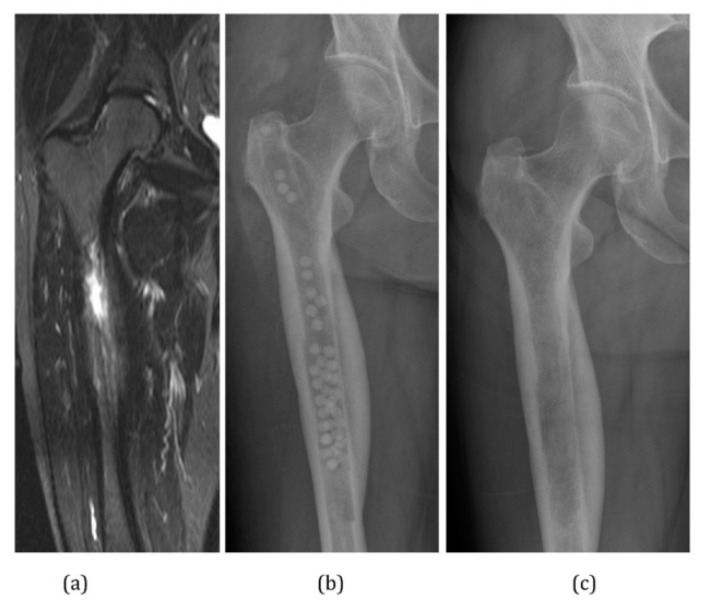
(**a**) MRI image demonstrating extensive medullary edema, intramedullary abscess, and cortical involucrum. (**b**) Infection treated by excision via medullary reaming. Dead space filled with calcium sulphate pellets loaded with gentamicin. Shown via radiograph (**c**) follow-up radiograph at 4 months post-operatively. Calcium sulphate pellets have dissolved completely. Reprinted from Ferguson et al. [68] following the Creative Commons Attribution (CC BY) license (http://creativecommons.org/licenses/by/4.0/) (accessed on 16 September 2021).

Biodegradable ceramics, such as calcium sulfate and calcium phosphate, are of strong interest for simultaneous use as bone void fillers and drug delivery vehicles in the clearance of bacterial osteomyelitis [68]. Calcium sulfate has been used in bone grafting since 1892 [68,146], and has a compressive strength equal to that of cancellous bone [68]. It is relatively inexpensive and is commercially available as hard pellets and liquid grafts [147]. Additionally, calcium sulfate possesses a quick resorption time, with a range of 3–12 weeks, depending on application (Figure 3) [68,146,147]. Jackson et al. [41] reported on calcium sulfate pellets loaded with amikacin, gentamicin, or vancomycin in vitro and found that the pellets eluted the antibiotics and dissolved completely within 16 h. These rapidly dissolving pellets loaded with amikacin were studied in an in vivo goat model by Bransetter et al. [89]. In that model, the pellets dissolved completely within 12 h and eluted amikacin above the MIC of *Pseudomonas aeruginosa* in 4–8 h. This type of pellet may be useful as part of a multimodal management plan for traumatic or contaminated wounds to provide rapid bacterial decontamination of an area, but this treatment is unlikely to be able to provide sustained antibiotic delivery. McKee et al. [148] demonstrated the use of calcium sulfate pellets as a dual platform device, delivering tobramycin and promoting bony union in cases of infected long bone non-unions in a prospective clinical trial. The experimental device was found to be particularly helpful to eliminate dead space and deliver antimicrobials while biodegrading. This study reported a 92% success rate (determined by clearance of osteomyelitis and creation of bony union), and a rate of 8% infection recurrence and drainage of antibiotic-rich fluid after pellet dissolution. Maale et al. [29] investigated the ability of a purified calcium sulfate preparation loaded with tobramycin and vancomycin to inhibit biofilm formation without stimulating systemic toxicity in 50 patients undergoing revision arthroplasty for infected total joints or after multiple major revisions. The patients demonstrated significant local antibiotic concentrations in the first five post-operative days and noted no persistent wound drainage, as has previously been described [148].

Clinically, calcium sulfate is not used as frequently as calcium phosphates for bone regeneration because of its rapid absorption, rapid loss of mechanical strength [149], and propensity to produce a serous discharge, which is thought to be a result of acidic byproducts during material degradation [68]. Based on the available literature, calcium sulfate may have a place in the treatment of bacterial osteomyelitis when rapid antimicrobial delivery, the ability to encourage bone development, and rapid degradation of the DDD are appropriate. As such, calcium sulfate is not an ideal single-system local DDD.

#### 3.3.2. Calcium Phosphate

Calcium phosphates have been used since the 1980s to enhance the osseointegration of metal implants [150] and are currently a popular synthetic graft substitute, as their chemical structure is similar to the mineral stage of bone [17,151]. These characteristics open a window of possibility into enhanced tissue regeneration, as they provide biocompatibility, bioactivity, and strong osteoconductive properties [95,150,152]. Calcium phosphates have a range of biodegradation profiles and mechanical properties, which are dictated by the calcium to phosphate ratio of the material [17,150]. The most commonly investigated CaP ceramics include HA, TCP, β-TCP, and dicalcium phosphate [17,68,152]. These CaP ceramics are typically in the forms of scaffolds, granules, cements, and coatings [95,150,152]. Additional benefits when using a CaP cement include an isothermic setting reaction, which allows a wider antimicrobial selection [17,149] than that of PMMA cement, and increased stimulation of angiogenesis when incorporating HA [17].

While there are strong benefits to CaP ceramics, there are also challenges. Challenges include inadequate mechanical strength, especially if needed in load-bearing portions of the skeleton [95,152]; small pore size and limited interconnectivity of pores, which can limit bone ingrowth and impact drug loading and release [95,150,153]; incongruities in material degradation and bone regeneration rates [95,154]; as well as the risk of bacterial colonization of slowly degrading ceramics [17]. Current investigations in the field of CaP ceramics have been centered around improving porosity [95,154,155,156], improving mechanical strength [95,156], and encapsulating drugs and growth factors into the CaP carrier [95]. Investigations into porosity of CaP ceramics have concluded that biodegradable poly (lactic-co-glycolic) acid (PLGA) microspheres are suitable for incorporation into CaP cements to increase porosity. PLGA microspheres typically degrade prior to the rest of the matrix, creating macropores that facilitate bone ingrowth and implant fixation [155,156], and may make the ceramic less brittle and, consequently, more appropriate for clinical use [157]. Duan et al. [154] found that the incorporation of PLGA microspheres into a CaP cement had appropriate osteoconductive properties, biodegradation, and good biomechanical properties in an in vivo rabbit model. An alternative approach to modifying porosity resulted in an injectable CaP drug delivery foam, which was produced and evaluated in vitro [153]. This drug delivery foam was capable of providing consistent release of bioactive doxycycline with potential for 3–4 weeks of sustained delivery in an in vitro system. This original investigation is an intriguing option for drug delivery in non-load bearing situations and warrants in vivo investigation. The works of Gonzalez-Sanchez et al. [158] and Bastari et al. [159] highlight the role of CaP films and coatings in nanomedicine and encapsulation of drugs for the clearance of osteomyelitis and enhancement of bone regeneration.

Ultimately, CaP materials provide many attractive qualities, including isothermic setting reaction, suitability for use with a wide range of antimicrobial compounds, and the ability to form strong bone–material interfaces. These characteristics certainly lend themselves towards use as a dual platform device for the management of bacterial osteomyelitis. Limitations are currently focused on variation and unpredictability in release kinetics, risk of suboptimal biocompatibility, and difficulty in large-scale production of a universally appropriate system [149]. With this in mind, CaP materials are of interest for use in individual situations, but each system deserves careful in vitro and in vivo investigation before commonplace clinical usage for local drug delivery and bone regeneration.

#### 3.3.3. Alumina

**Figure 4:**
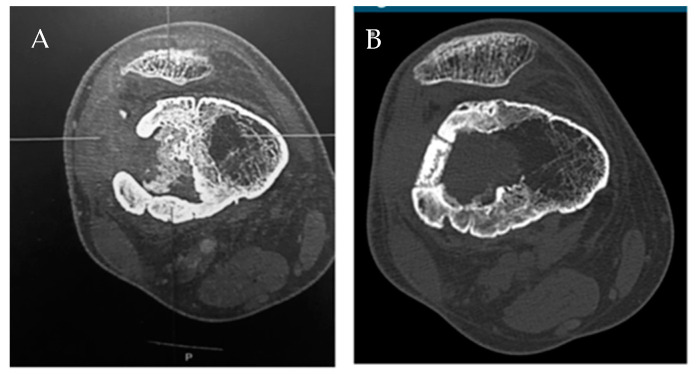
Image (**A**): pre-operative axial CT scan of the femur. Bone loss and bone remodeling as a result of chronic infection (osteomyelitis) is seen. Image (**B**): axial CT scan after follow-up of 11 months. Tight contact between bone and porous alumina ceramic is seen and demonstrates appropriate biocompatibility and osseointegration. Reprinted from Fiorenza et al. [144] following the Creative Commons Attribution (CC BY) license (http://creativecommons.org/licenses/by/4.0/) (accessed on 16 September 2021).

Alumina is an inert substance with good wear properties and is used frequently in artificial joint replacements and dental applications [160] owing to good biocompatibility and compressive strength [161]. Porous alumina is also clinically utilized in the fabrication of orbital implants following enucleation with the goal of allowing a fibrovascular network ingrowth through the device [152]. However, porous alumina has also been investigated as a drug delivery scaffold for clearance of bacteria and prevention of bacterial colonization [144,161,162]. Alumina-based ceramics are described to completely release loaded antibiotics and successfully resist bacterial colonization and biofilm formation in vivo [161]. Fiorenza et al. [144] describe a case of chronic osteomyelitis caused by *S. aureus* that underwent a successful one-stage surgical procedure using gentamicin-loaded porous alumina ceramic. The precise size and shape of the ceramic were determined via pre-operative CT scan (Figure 4), and the customized ceramic was loaded with gentamicin, as selected by culture and sensitivity. Follow up of greater than 14 months post-operatively indicated resolution. Similar positive results were reported by Denes et al. [162], who reported two patients affected by mediastinitis resulting in destruction of the sternum, and one patient with an infected ankle arthroplasty. These patients were treated with antibiotic loaded porous alumina in a one-step surgical procedure made possible by the compressive strength of alumina and were reported to remain infection-free at follow-up over 12 months post-operatively. This material and procedure represent a promising option for treatment of osteomyelitis that involves bone loss and allows for pre-operative imaging. Particular strengths of this system include the surface resistance to bacterial colonization, which could greatly reduce the risk of persistent bacterial infection, and the capability for a single surgical intervention, as opposed to multiple revision procedures.

#### 3.3.4. Bioactive Glass

Bioactive glass is a synthetic silica-based material [163] that was initially developed in the 1960s [145,164] and has been used clinically since 1985 [152,165]. The main benefits of bioactive glasses include bioactivity, osteoconductive and osteoinductive properties, biodegradation [152,166], load bearing capabilities [163], and the ability to create a local environment that is hostile to microbial growth [113,163]. In fact, bioactive glass is said to produce higher quantity and quality of bone when compared with synthetic HA [167] and newer hybrid materials have tailorable degradation, which is attractive [168]. Bioactive glasses have become a focus of investigations for drug delivery within the last one to two decades [166,169,170] because of their strong regenerative qualities, reported biocompatibility [171], and the initial experimental use of bioactive glasses in the treatment of chronic osteomyelitis [172]. Hasan et al. [167] created a biodegradable, bioactive glass-based antibiotic-releasing putty designed to be press-fitted into bone defects to provide support for bone growth while delivering antimicrobials (vancomycin) for 4–6 weeks to combat bacterial osteomyelitis. This material demonstrated vancomycin elution above the MIC of *S. aureus* for over 6 weeks in vitro, and as a putty, is attractive to surgeons because it can be formed into various dimensions [168]. Similarly, Soundrapandian et al. [166] found that a gatifloxacin-loaded bioactive glass loaded released gatifloxacin for up to 6 weeks in vitro. Drug release was influenced by scaffold size, concentration of drug solution, polymer coat, and dissolution medium. These results, along with the advantageous bioactive properties of bioactive glasses, leave them as an interesting multifunctional device for treatment of bacterial osteomyelitis. The current challenges lie within the optimal composition and fabrication process of bioactive glasses [171]. However, with a scalable process, it is likely that these materials will be further investigated for drug delivery in the treatment of bacterial osteomyelitis.

### 3.4. Polymers

#### 3.4.1. Natural Polymers

Natural polymers are of interest in tissue engineering and drug delivery as a result of their bioactivity, biocompatibility, and biodegradation. These qualities are accompanied by risks of immunogenicity, as well as poor mechanical properties, which limit their use in load-bearing situations [113,118]. Natural polymers, such as collagen, fibrin, and chitosan, are commonly utilized in a wide array of applications including attempted creation of blood vessels [173] and local delivery of antibiotics in many soft tissue and orthopedic applications [69,174]. Collagen is of particular interest, as it is a key component of the extracellular matrix [69,113]. Investigations into collagen and fibrin gels as DDDs have typically reported a rapid burst release in vitro, with approximately 90% of the antibiotic released during the first day and complete elution occurring by the fourth day [69]. Chitosan gels and sponges have been reported in vitro to produce a sustained antimicrobial release sufficient to inhibit *S. aureus* growth over the course of three weeks [175]. These gels have potential to be used as coatings to minimize the initial burst release of antibiotics [69]. Chitosan carries the additional intrigue of intrinsic antibacterial properties [176]. The role of these natural materials in the clearance of bacterial osteomyelitis is most likely strongest as adjunctive therapy either in acute infection, situations where mechanical stabilization is provided by other materials, or as coatings upon other materials to prolong antimicrobial delivery.

#### 3.4.2. Synthetic Polymers

Synthetic polymers are an area of extreme interest for use in local DDDs [113] for the clearance of chronic bacterial infection and bacterial osteomyelitis [69]. These materials are attractive because of their general biocompatibility, biodegradation, and versatility [113,118], which includes tunable drug release kinetics and degradation rates [69]. Synthetic polymers also have more controlled manufacturing methods, which lends confidence to quality control and lessens concerns of immunogenicity and imperfections [69,113,177,178]. Similar to natural polymers, synthetic polymers possess less than ideal mechanical properties, which leaves them inadequate for independent use in load-bearing areas [113,118,177]. Additionally, the degradation of synthetic polymers most often occurs via hydrolysis, which can create an acidic pH in tissue adjacent to the implant, resulting in inflammation, local tissue damage, and potential alteration of local antimicrobial efficacy [69,113,169]. There are many polymers that are of interest, including PLGA, one of the most well investigated polymers [169], as well as polyurethane (PUR), poly(lactic acid) (PLA), poly(glycolic acid) (PGA), and poly(caprolactones) (PCL) [22,69,113]. McLaren et al. [22] investigated the ability of an injectable PLGA modified with plasticizer polyethylene glycol (PEG) and antibiotics (gentamicin and clindamycin) to prevent bacterial infection and facilitate new bone growth in an in vivo ovine contaminated bone defect. This material was found to release about 50% of its antibiotics within the first seven days of elution and was effective at preventing persistent bacterial infection in this model. Additionally, less than 1% of the loaded antibiotics were present three weeks after implantation, which suggests this system may offer a lower risk of antimicrobial resistance than PMMA, which has been found to release subtherapeutic levels of antibiotics even five years after implantation [14]. Alongside investigations of pure polymers, there are innumerable options for composite polymers, which are an area of interest and promise in drug delivery and tissue regeneration [113]. Recently, a composite polymeric scaffold was reported to be an effective delivery platform for delivery of antibiotics for the elimination of *S. aureus* from contaminated bone defects in rabbits. This scaffold was purported to serve the dual role of tissue regeneration scaffold and antibiotic delivery device [105].

Synthetic polymers provide a seemingly endless supply of materials and composites to engineer biocompatible, biodegradable devices that deliver drugs and regenerate bone. Extensive options are, of course, accompanied by the need for extensive investigation and characterization before commonplace clinical usage of any individual polymer system. It is highly likely that synthetic polymers will continue to provide clinically relevant results in the management of bacterial osteomyelitis.

### 3.5. Emerging Nanotechnology for Combatting Bacterial Infection

Nanotechnology, with an emphasis on nanopatterning and nanoparticles, has emerged as an area of incredible interest in simultaneous stimulation of tissue regeneration, prevention of bacterial infection, and mitigation of antimicrobial resistance via intrinsic device properties and drug delivery [42,179,180,181,182].

#### 3.5.1. Nanopatterns

Nanopatterning refers to the micro and nano-scale surface features that are either inherent to surfaces, such as nanopillars on cicada wings [183], various nanotextures on plants, lizards, and sharks [180], or inspired by nature and carefully engineered in a laboratory [54,184]. Nanopatterns provide a multitude of applications, from the ability to kill adherent bacteria [184] to the ability to determine stem cell fate [181]. While mechanisms of bactericidal action are not completely elucidated, proposed hypotheses are typically centered around mechanical forces [54], including the stretching, puncturing, and eventual rupture of bacterial cells [54,96,185]. These hypotheses acknowledge the complex dynamics between various bacterial cells and nano-patterned surfaces and have recognized factors such as the presence of cellular motility [96], cellular morphology (e.g., rod-shaped vs. coccoid) [186], cell wall components and structure (Gram-positive vs. Gram-negative) [187], and extracellular polymeric substance (EPS) [185], as important in nanopattern–bacterial cell interactions. The main benefits of utilizing nanopatterns for intrinsic antibacterial activity include elimination of antimicrobial agents from surface coatings and delivery vehicles. Nanopatterns can mitigate the risks of subtherapeutic levels of antimicrobials and propagation of antimicrobial resistance, especially in the face of biofilms, which can harbor bacterial cells that are 10–1000-fold less susceptible to antimicrobials than planktonic bacteria [184,188,189,190]. Additionally, nanopatterns may enhance biocompatibility by bypassing the need for chemical surface modifications [184].

**Figure 5:**
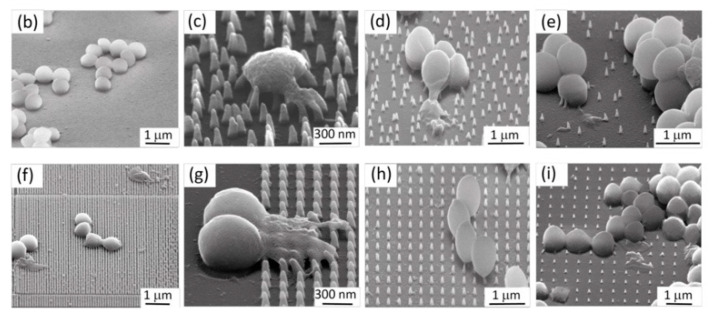
SEM images of *S. aureus* on (**b**) control surface and (**c**–**i**) various experimental surfaces. Damaged bacterial cells can be identified by an irregular morphology (**c**–**i**) compared with healthy cells in typical coccoid morphology (**b**). (**f**) Nanopillars with interspace of 100 nm and (**g**) nanopillars with interspace of 170 nm demonstrated the most efficient bactericidal properties. Reprinted from Modaresifar et al. [54] following the Creative Commons Attribution (CC BY) license (http://creativecommons.org/licenses/by/4.0/) (accessed on 16 September 2021).

Dickson et al. [184] utilized a scalable process of soft and nanoimprint lithography to imprint nanopillars onto PMMA films to investigate a potentially broadly bactericidal surface pattern targeted against *Escherichia coli* (*E.coli*), which is a leading cause of Gram-negative orthopedic implant infections [191]. Smaller, more closely spaced pillars were more effective, possibly owing to greater stresses as a result of the bacteria contacting more pillars simultaneously. Michalska et al. [180] systematically investigated bactericidal activities of a variety of surfaces with three Gram-negative bacterial species (*Escherichia, Pseudomonas*, and *Rhodobacter*) and a Gram-positive *Bacillus.* They also observed two obvious mechanisms; one being that longer, sharp pillars were able to directly pierce microbial cells, nonselective of species. The second mechanism being that shorter, blunt pillars required multifaceted cellular interactions to eventually stretch and tear membrane envelopes. The effects of interspacing and controlled disorder on the functionality of a specific bactericidal nanopattern were investigated by Modaresifar et al. [54] utilizing *S. aureus*, as the most common pathogen in implant-associated infections. This study quantified numbers and characterized the morphology of *S. aureus* cells on nanopatterns via scanning electron microscopy (SEM) (Figure 5) and determined, similarly to Dickson et al. [184], that decreased interspacing (100 nm) demonstrated the greatest bactericidal efficiency and that controlled disorder did not enhance bactericidal efficiency. Similar to other reports, the main bacterial killing mechanism was direct penetration of the cell wall and eventual rupture. Widyaratih et al. [181] investigated the antibacterial behavior of multiple types of osteogenic nanopatterns using a strain of *E.coli*. Nanopillars were created on silicon wafers using an electron beam-induced deposition (EBID) system. The results confirmed previous work that controlled nanopatterns can be produced by EBID and indicated that nanopatterns containing features of interspace and controlled disorder, which are derived from osteogenic nanopatterns, could exhibit bactericidal properties against *E. coli*.

These investigations spark interest into the creation of controlled, reproducible nanopatterns to serve in a dual platform functionality, for tissue regeneration and prevention of bacterial infection, by instructing mesenchymal stem cells to commit to osteogenic lineage and exerting bactericidal effects [181]. Dual platform functionality could result in profound nanomanufacturing to prevent biofilm formation on the surfaces of a wide range of implantable devices. The potential to create nanopatterns on materials appropriate for orthopedic use, such as titanium and polymers [181], is fascinating and unlocks an incredibly promising area of exploration. This area holds exceptional promise in orthopedic procedures and the prevention and treatment of bacterial osteomyelitis.

#### 3.5.2. Nanoparticles

Nanoparticle delivery systems are based on magnetic nanoparticles (MNPs), which are described as a class of <100 nm engineered materials, typically composed of iron, nickel, cobalt, and their oxides, that can be manipulated by an applied external magnetic field [192]. Nanoparticle delivery systems offer many advantages, including immune system evasion, the ability to modulate drug release kinetics and target drugs to specific sites, improved multi-drug delivery [42], and potential bacterial detection [193]. Various combinations of MNPs and antibiotics have been investigated for the ability to penetrate bacterial cells and biofilms as a method to render bacteria inactive [193], and there are many investigations into general antibacterial strategies, as well as strategies specifically geared towards bacterial osteomyelitis.

Geilich et al. [189] developed a multi-compartment polymersome formulation that contains hydrophobic superparamagnetic iron oxide nanoparticles (SPIONs) and hydrophilic methicillin that is biocompatible and intended for the treatment of medical device-associated infections. The efficacy of this system was assessed in an in vitro methicillin-resistant *Staphylococcus epidermidis* biofilm. The results indicated that this system of SPIONs, co-encapsulated with antibiotics, was able to eliminate the biofilm in vitro via direct application and external magnetic stimulation. Akram et al. [194] investigated a triple combination therapy of silver magnetite nanoparticles (AgNPs) with blue light and either amoxicillin, azithromycin, clarithromycin, linezolid, or vancomycin against 10 clinical isolates of MRSA in vitro. This work is interesting, as it combines the antibacterial activity that AgNPs have been said to promise [195,196] with the efficacy of blue light against MRSA and the properties of conventional antibiotic therapy. The results indicated enhanced bactericidal activity of AgNPs applied in combination with blue light and found that bactericidal activities were greatest when either clarithromycin or azithromycin was included in the triple therapy. This triple combination therapy presents an intriguing novel approach to combatting MRSA infections while reserving last-line therapies such as vancomycin, although honing for clinical use is definitely warranted.

There are multiple investigations into nanoparticle systems for the treatment of bacterial osteomyelitis designed to overcome the shortcomings of current therapies [158,190,197,198,199]. Posadowska et al. [197] suggested the use of a vancomycin-enriched injectable gellan gum (hydrogel) matrix. Enrichment was accomplished by both dissolved vancomycin and vancomycin encapsulated in PLGA nanoparticles. The results from in vitro studies indicated relatively simple and precise dosing of the hydrogel, a prominent burst release of vancomycin followed by a prolonged, sustained delivery that was thought to be due to a combination of erosion-diffusion release, appropriate antimicrobial activity against *Staphylococcus* sp., and cytocompatibility with osteoblast-like cells. Similarly, Gonzalez-Sanchez et al. [158] developed an acrylate multifunctional orthopedic hydrogel that was reported in vitro to be osteoconductive, and possessed antibacterial effects as a result of silver nanoparticle adsorption.

Qiao et al. [198] report on the use of Fe_3_O_4_ nanospheres combined with functionalized carbon nanotubes (CNTs) and gentamicin in conjunction with a combined microwaveocaloric-chemotherapy (MCCT) system for dual-targeting and microwave (MV)-excited drug release for the clearance of MRSA-induced osteomyelitis. This study found that Fe_3_O_4_/CNT/gentamicin had excellent bacteria-capturing capabilities in vitro, and demonstrated bacterial load reduction in an in vivo rabbit model of osteomyelitis. The proposed mechanism of action of this system is complex. It is thought to be initiated with a synergistic reaction of the synthesized nanocapturer binding bacteria, which then produces heat under MV stimulation and triggers the release of gentamicin. In a fairly similar fashion, Fang et al. [190] investigated the heating effect of MNP-induced hyperthermia to both destroy biofilm and promote antibiotic efficacy to improve the treatment of peri-implant osteomyelitis. An in vivo rat model was created by implanting a metallic needle with or without bacterial contamination into the intramedullary canal of the femur. Fe_3_O_4_ nanoparticles were utilized in combination with intramuscular injection of vancomycin +/− magnetic hyperthermia. Colony forming units (CFUs) and histology indicated that the combination of MNPs and hyperthermia could destroy the experimental biofilm and enhance the overall therapeutic effect of systemic and local therapy.

Lastly, Ak et al. [199] developed a novel biodegradable, biocompatible, physically targeted gentamicin-loaded gelatin nanoparticle system for the local treatment of bacterial osteomyelitis. Through an in vivo rat model of proximal tibial *S. aureus* osteomyelitis, the drug delivery system was shown to hasten the recovery time of infected rats in comparison with free gentamicin or placebo therapy. Additionally, this system demonstrated controlled drug release in vitro and warrants further investigation into use for the treatment of osteomyelitis.

Ultimately, the works described above represent novel endeavors into the treatment of implant-associated biofilms, bacterial infection, and osteomyelitis. These investigations have the potential to mitigate many of the challenges that face current local drug delivery and tissue regeneration systems. As is common with nanotechnologies, there may be challenges associated with upscaling the proposed systems. Additionally, detailed investigations into safety are necessary prior to clinical translation, as it is well understood that MNP systems carry risks of in vivo toxicity resultant from nanoparticle composition [200] as well as accumulation within the body [201].

## 4. Antimicrobial Strategies

When pursuing systemic antimicrobial therapy, as well as local antimicrobial delivery, there are many considerations, including bactericidal versus bacteriostatic and time- versus concentration-dependent antibiotics [202]; the choice to use single-agent or combination therapy [101]; along with concerns of patient sensitivity, compliance, and adverse effects [203]. Local drug delivery can mitigate the severity of systemic toxicity and adverse effects, but local drug delivery is accompanied by considerations of the feasibility of drug incorporation into the DDD. While this review does not focus on specific antimicrobial strategies, the authors recommend the following references for those readers interested in learning more about antibiotics for local drug delivery: [101,203,204,205].

## 5. Conclusions and Future Perspectives

Bacterial infection is a serious complication following surgical implantation of medical devices and may occur shortly post-operatively or months to years later. Current therapies of systemic antimicrobials, surgical debridement, and local drug delivery are imperfect. Chronic, persistent, and recurrent bacterial infection still plague this patient population. Certainly, there are steps being taken to reduce the risk of post-operative bacterial infections including osteomyelitis. These steps include remaining cognizant of the risk for multidrug-resistant bacterial organisms; providing patient education and encouraging patients to cease smoking in the weeks leading up to scheduled procedures; maintaining appropriate and stable blood glucose and body temperature; and maintaining exceptional skin, body, and wound hygiene peri-operatively [33,35,47]. Despite these measures, post-operative bacterial infection will still occur and, therefore, the needs for effective therapies remain. While there is significant progress being made in the field of multifunctional devices for tissue regeneration and drug delivery, progress is slow, as these devices are quite specific to their intended application, and persistent challenges remain. Challenges include adverse host reaction to the material, insufficient antimicrobial delivery, biofilm formation on the device, as well as insufficient mechanical properties and mismatched degradation and regeneration profiles.

Further investigations into engineering materials that possess the ideal characteristics for a drug delivery device, including biocompatibility, clinically significant and controlled release of drugs, predictable and inert degradation, and appropriate mechanical characteristics, will continue to provide new and improved therapies. During these pursuits, it is important to keep cost in mind. There are many challenges in scaling the production of devices from small batches to more readily available products, and product cost may influence the provider’s decisions to utilize certain products. Recent novel discoveries and approaches, such as porous alumina for simultaneous drug delivery and mechanical support, and modifications and novel combinations within the class of synthetic polymers offer tremendous potential for future cures to the prevention and treatment of these debilitating infections. Perhaps one of the most intriguing and promising opportunities is the field of nanotechnology. Nanotechnology offers promise for minimally invasive and local therapies that are bactericidal, regenerative, and stimuli-responsive and may minimize the risks of developing antimicrobial resistance, biofilm formation, and device failure. Lastly, there are many promising devices and investigations. Perhaps an ideal way to approach the persistent, significant problem of bacterial osteomyelitis is to acknowledge that there may not be one single ideal device. Instead, there are multiple appropriate options for various clinical scenarios.

## Data Availability

Not applicable.
